# Risk factors and control of *Opisthorchis viverrini* in the Lower Mekong Basin: A systematic review

**DOI:** 10.1371/journal.pntd.0013790

**Published:** 2025-12-11

**Authors:** Suji Y. O’Connor, Mary Lorraine Mationg, Matthew J. Kelly, Callum Lowe, Gail M. Williams, Archie C.A. Clements, Banchob Sripa, Somphou Sayasone, Virak Khieu, Kinley Wangdi, Donald E. Stewart, Sirikachorn Tangkawattana, Apiporn T. Suwannatrai, Vanthanom Savathdy, Visal Khieu, Sean N. Gannon, Peter Odermatt, Catherine A. Gordon, Sangduan Wannachart, Donald P. McManus, Darren J. Gray

**Affiliations:** 1 National Centre for Epidemiology and Population Health, Australian National University, Acton, Australian Capital Territory, Australia; 2 QIMR Berghofer, Herston, Queensland, Australia; 3 School of Public Health, University of Queensland, Herston, Queensland, Australia; 4 School of Biological Sciences, Queen’s University Belfast, Belfast, United Kingdom; 5 Department of Tropical Medicine, Faculty of Medicine, Khon Kaen University, Khon Kaen, Thailand; 6 Tropical Disease Research Center, Faculty of Medicine, Khon Kaen University, Khon Kaen, Thailand; 7 Lao Tropical and Public Health Institute, Ministry of Health, Vientiane, Lao People’s Democratic Republic; 8 National Center for Parasitology, Entomology and Malaria Control, Ministry of Health, Phnom Penh, Cambodia; 9 HEAL Global Research Centre, Health Research Institute, Faculty of Health, University of Canberra, Bruce, Australian Capital Territory, Australia; 10 School of Medicine and Dentistry, Griffith University, Nathan, Queensland, Australia; 11 Faculty of Veterinary Medicine, Khon Kaen University, Khon Kaen, Thailand; 12 Department of Parasitology, Faculty of Medicine, Khon Kaen University, Khon Kaen, Thailand; 13 Swiss Tropical and Public Health Institute, Allschwil, Switzerland; 14 University of Basel, Basel, Switzerland; 15 Centre for Tropical Health and Emerging Diseases, QIMR Berghofer, Herston, Queensland, Australia; LSTM: Liverpool School of Tropical Medicine, UNITED KINGDOM OF GREAT BRITAIN AND NORTHERN IRELAND

## Abstract

**Background:**

*Opisthorchis viverrini* (OV) is a major public health concern in the Lower Mekong Basin. This study aimed to synthesize all field-based empirical research examining risk factors and control strategies for OV in the Lower Mekong Basin (LMB).

**Methods:**

We performed a systematic review of published literature (1990–2024) on field-based OV studies that examined risk factors and control strategies in LMB. The literature search included two databases: PubMed and Scopus. We included field-based studies that analysed or reported on OV risk factors or control strategies using quantitative or mixed methods and were written in English. We excluded secondary research articles, laboratory-based research, qualitative only research and studies conducted outside LMB. All prospective studies underwent quality assessment using the Newcastle-Ottawa Scale or the Cochrane Risk of Bias tool II prior to final inclusion.

**Results:**

We identified 807 citations from PubMed and Scopus. From those, 56 studies were included in the review and three additional studies were identified from citation searches of included studies in the review. Studies were extracted and analysed by research focus. Among the included studies, 45 were conducted in Thailand, 11 in Laos, two in Vietnam, and one in Cambodia. Factors associated with OV infection were explored in 51 studies, and 11 studies reported on control strategies. General education was found to be an important protective factor for OV infection. Consumption of raw or undercooked fish was the most reported risk factor. Anthelmintic treatment was the primary control strategy across studies.

**Conclusion:**

This review summarises risk factors and control strategies reported in LMB since 1990. We found that sociodemographic, environmental, and economic factors were important predictors of OV infection. Given the multitude of risk factors for infection identified in this study and the complex lifecycle of OV, we recommend a One Health approach, that recognises the interconnectedness of human, animal and environmental health, for future health promotion and control strategies.

**Trial registration:**

PROSPERO registration ID: CRD42022357080.

## Author summary

*Opisthorchis viverrini* (OV) is a liver fluke that is causally linked to cholangiocarcinoma, a type of bile duct cancer that is often fatal. OV is endemic to the Lower Mekong Basin –a region in Southeast Asia that includes Northeast Thailand, Lao People’s Democratic Republic, Southern Vietnam, and Cambodia– and is estimated to affect over 12 million people in the region. We conducted a systematic review of all field-based literature on OV risk factors and control strategies in the Lower Mekong Basin. The majority of studies were conducted in Thailand. Our review found that consumption of raw or undercooked fish was the most reported risk factor for OV and anthelmintic treatment with praziquantel was the primary control strategy. Our findings can be useful for informing future OV target areas for disease education and prevention.

## Background

*Opisthorchis viverrini* (OV), a parasitic liver fluke, is a major public health concern in mainland Southeast Asia [1]. Chronic OV infections are causally associated with cholangiocarcinoma (CCA), a severe and often fatal bile duct cancer [2–4]. It is estimated that 12.39 million people are impacted by OV, with the highest burden in the Lower Mekong Basin (LMB) [5], a region that includes Thailand, Lao People’s Democratic Republic (henceforth Laos), Cambodia, and Vietnam. In 2023, reported OV prevalence in Cambodia ranged from 64.7% to 65.5% [6]. In Thailand, a 2019 national survey reported 2.2% OV prevalence, with the highest regional prevalence in northeast Thailand (4.97%) [7]. A 2016 study reported 68.0% prevalence in one district of Kalasin Province, Northeast Thailand [8]. Similar studies reported high OV prevalence in Laos [9,10], reaching 87.9% in studied communities in Khammouane Province in 2015 [10]. Binh Dinh Province in Vietnam has been found to be endemic for OV, with 11.4% prevalence in 2015 [11].

**Fig 1:**
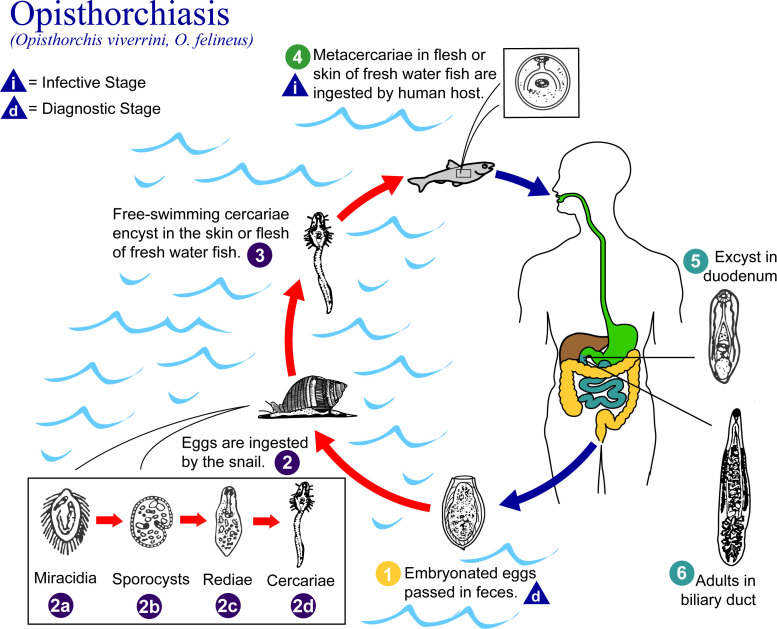
Opisthorchiasis lifecycle. (Source: CDC, 2002. Alexander J. da Silva, PhD; Melanie Moser, https://www.cdc.gov/dpdx/opisthorchiasis/index.html).

Acute or mild OV infections can cause gastrointestinal symptoms such as constipation, abdominal pain and diarrhoea, but are often asymptomatic and thus can evade detection [12]. Higher intensity and chronic infections can cause inflammation of the bile ducts and surrounding areas, obstructive jaundice and other biliary disorders, which in turn increase risk of CCA [13]. The mainstay of OV control is mass drug administration of praziquantel (PZQ) [14], although hepatobiliary abnormalities can persist after treatment [13]. The difficulties of OV detection and treatment are further complicated by OV’s multistage lifecycle involving two intermediate hosts (aquatic snails, cyprinid fish) and one definitive host (humans and other fish-eating mammals), as shown in Fig 1 [12,15]. While animal research is limited, studies in Northeast Thailand reported 11.9% prevalence in cyprinid species in 2022 [16], and 47.6% in non-human mammalian hosts in 2018 [15].

To control and prevent OV, a multifaceted approach is needed. ‘One Health’ is an integrated health approach that recognises the interconnectedness of human, animal and environmental health [17]. Using a ‘One Health’ approach, this review seeks to synthesise (1) risk factors and protective factors for OV infection, and (2) effectiveness of existing control strategies.

## Methods

The protocol for this systematic review was registered in the PROSPERO database under registration number (CRD42022357080).

The Preferred Reporting Items for Systematic Reviews and Meta-Analyses (PRISMA) [18] were followed for reporting systematic reviews in this article (see S1 Table).

### Search strategy and selection criteria

Searches were performed on 02 September 2022 and updated on 13 September 2024. We searched PubMed and Scopus online databases for empirical field studies on OV (for specific search terms, see S1 Text). Studies were eligible if they examined OV risk factors or control strategies, were empirical field-based research in the LMB and were published in English after 1990. Studies were excluded if they took place outside the LMB or relied on secondary data or laboratory research (see Table 1).

**Table 1:** Study inclusion and exclusion criteria.

Category	Inclusion	Exclusion
**Admissible studies**	Field studies that examined risk factors or control strategies conducted in the Lower Mekong region	Secondary data Laboratory research Field studies that solely examined OV burden Any field study that was conducted outside the Lower Mekong region
**Study design**	Cross-sectional Cohort Case-control Experimental design Observational studies	Systematic literature review Scoping review Meta-analysis Qualitative studies
**Population**	All human and animal populations in the Lower Mekong Basin	Populations that resided outside of the Lower Mekong Basin
**Time period**	Published 1990 onwards	Published before 1990
**Outcomes**	Knowledge, attitudes and practices Risk factors Drug efficacy	Non-OV health outcomes
**Quality assessment**	Some concerns Low risk of bias Moderate quality High quality	High risk of bias Low quality
**Publication language**	English	Non-English languages

Identified studies were catalogued in an Endnote library (Version X.9) for de-duplication and removal of studies published before 1990. Studies were then imported to Rayyan [19] and were screened by two independent reviewers (SYO, CL) at the title, abstract and full-screening stage. Conflicts were resolved by discussion between reviewers. In cases where a study was reported in more than one publication, the publication with the most detailed information was retained. All included studies were extracted into Microsoft Excel using a standardised form. Data were grouped by country, year, study design, and research focus. Hand-searching of references was undertaken for all included studies, and relevant studies identified.

### Quality assessment

Quality assessment was undertaken for all eligible studies (see S2–S6 Tables). The Cochrane risk-of-bias tools were used for all experimental human trials and scored in accordance with existing criteria [20]. Trials determined to be “low risk” or “some concerns” were included in the review. The original Newcastle-Ottawa Quality Assessment Scale (NOS) [21] and the Herzog et al. adapted version [22] were used for non-experimental studies. We also adapted NOS for epidemiological and animal studies (S2 and S3 Texts). Scoring was developed based on quality thresholds devised by Hosking and colleagues [23]; to accommodate for sections that were non-applicable, scores were considered as follows: 0–39% low quality; 40–69% moderate quality, 70–100% high quality. Studies that received a “moderate” or “high” quality rating were retained in the review.

## Results

**Fig 2:**
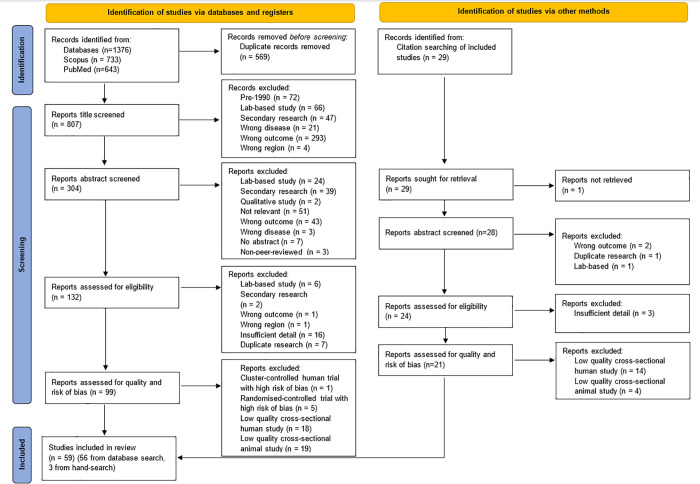
PRISMA 2020 flow diagram for systematic reviews [18].

Our database search identified 807 unique reports, of which 99 reports were deemed eligible following full-text screening (see S7 Table for excluded studies). Following quality assessment, 56 were retained. References of all included studies were then hand-searched for eligible reports and three studies retained following eligibility and quality screening (see Fig 2).

In total, 59 empirical field studies on OV risk factors and control were included in the review. Studies were conducted in Thailand (n = 45), Laos (n = 11), Vietnam (n = 2) and Cambodia (n = 1). Cross-sectional design was most common (n = 43). Of the remaining studies, seven were experimental, one was quasi-experimental, six were cohort studies and two were case-control (see Table 2).

**Table 2:** Characteristics of included studies.

	Country	Population size	Study design	Diagnostic method	Prevalence^*^	Research focus	Quality assessment^**^
**Araki et al (2018) [10]**	Laos	348 humans	Cross-sectional	Kato-Katz	87.9%	Risk factors	Moderate
**Aunpromma et al (2012) [24]**	Thailand	214 cats 821 dogs	Cross-sectional	FECT	Cats = 35.51% Dogs = 0.37%	Risk factors	Moderate
**Aunpromma et al (2016) [25]**	Thailand	1018 dogs 239 cats	Cross-sectional	Microscopy	Cats = 30.9% Dogs = 0.2%	Risk factors	Moderate
**Bukkhunthod et al (2020) [26]**	Thailand	80 humans	Cluster-RCT	N/A	N/A	Control strategies	Some concerns
**Chaiputcha et al (2015) [27]**	Thailand	254 humans	Cross-sectional	Modified Kato-Katz	14.96%	Risk factors	Moderate
**Charoensuk et al (2022) [16]**	Thailand	225 humans 778 cyprinid fish	Cross-sectional	FECT, cercarial shedding	Humans = 7.1% Fish = 11.9%	Risk factors	Moderate
**Charoensuk et al (2024) [28]**	Thailand	3600 humans	Cohort	Modified FECT	6.0%	Control strategies	High
**Chuangchaiya et al (2019) [29]**	Thailand	495 humans	Cross-sectional	Kato-Katz	15.4%	Risk factors	Moderate
**Chuangchaiya et al (2020) [30]**	Thailand	564 humans	Cross-sectional	Kato-Katz	12.9%	Risk factors	Moderate
**Chudthaisong et al (2015) [31]**	Thailand	526 humans	Case-control	N/A	N/A	Risk factors	Moderate
**Dao et al (2016) [11]**	Vietnam	254 humans	Cross-sectional	Kato-Katz	11.4%	Risk factors	Moderate
**Dao et al (2017) [32]**	Vietnam	1200 snails 754 fish	Cross-sectional	Cercarial shedding	*Bithynia siamensis goniomphalos *= 0.87% *B. funiculata* = 0.14% Fish = 45.0%	Risk factors	Moderate
**Forrer et al (2012) [33]**	Laos	3371 humans	Cross-sectional	Kato-Katz	61.1%	Risk factors	Moderate
**Haswell-Elkins et al (1991) [34]**	Thailand	486 humans	Cross-sectional	FECT	69.9%	Control strategies	Moderate
**Kaewpitoon et al (2016) [35]**	Thailand	978 humans	Cross-sectional	Kato-Katz	1.74%	Risk factors	Moderate
**Kaewpitoon et al (2012) [36]**	Thailand	1168 humans	Cross-sectional	Modified Kato thick smear and microscopy	2.48%	Risk factors	Moderate
**Kaewpitoon et al (2012) [37]**	Thailand	333 humans	Cross-sectional	Modified Kato thick smear	9.91%	Risk factors	Moderate
**Kaewpitoon et al (2016) [38]**	Thailand	730 humans	Cross-sectional	Kato-Katz	3.02%	Risk factors	Moderate
**Kaewpitoon et al (2016) [39]**	Thailand	355 humans	Cross-sectional	Modified Kato-Katz	2.25%	Risk factors	Moderate
**Kaewpitoon et al (2016) [40]**	Thailand	80 humans	Cross-sectional	Mini-Parasep solvent free concentration technique	1.25%	Risk factors	Moderate
**Kaewpitoon et al (2018) [41]**	Thailand	403 humans	Cross-sectional	FECT	11.9%	Risk factors	Moderate
**Kaewpitoon et al (2019) [42]**	Thailand	400 humans	Cross-sectional	Mini-Parasep solvent free concentration technique	0.25%	Risk factors	Moderate
**Kitphati et al (2021) [43]**	Thailand	9904 humans	Cross-sectional	Kato-Katz	4.0%	Risk factors	Moderate
**Kopolrat et al (2020) [44]**	Thailand	59727 snails	Cross-sectional (repeated)	Cercarial shedding	Snails = 0.71%	Risk factors	Moderate
**Laithavewat et al (2020) [45]**	Thailand	131 humans	Cluster-RCT	N/A	N/A	Control strategies	Some concerns
**Laoraksawong et al (2018) [46]**	Thailand	602 humans	Cross-sectional	FECT	19.4%	Risk factors	Moderate
**Lovis et al (2012) [47]**	Laos	93 humans	RCT	Kato-Katz	98.9%	Control strategies	Some concerns
**Miyamoto et al (2014) [48]**	Cambodia	16082 humans	Cross-sectional	Kato-Katz	7.66%	Risk factors	Moderate
**Moonsan et al (2024) [49]**	Thailand	36 humans	Cross-sectional	N/A	N/A	Control strategies	Moderate
**Nakbun et al (2018) [50]**	Thailand	134 humans	Cross-sectional	FECT	56%	Risk factors	Moderate
**Nithikathkul et al (2009) [51]**	Thailand	327 humans	Cross-sectional	FECT	5.2%	Risk factors	Moderate
**Ong et al (2016) [52]**	Thailand	632 humans	Cross-sectional	FECT	Nong Bua Lamphu = 5.45% Khon Kaen = 26.42%^#^	Risk factors	Moderate
**Padchasuwan et al (2018) [53]**	Thailand	1100 humans	Cross-sectional	N/A	N/A	Risk factors	High
**Panithanang et al (2018) [54]**	Thailand	66 humans	Quasi-experimental trial	N/A	N/A	Control strategies	Some concerns
**Phongluxa et al (2013) [55]**	Laos	574 humans	Cross-sectional	Kato-Katz	88.7%	Risk factors	Moderate
**Prachaiboon et al (2021) [56]**	Thailand	1163 humans	Cross-sectional	N/A	N/A	Risk factors	Moderate
**Prakobwong & Suwannatrai (2020) [57]**	Thailand	3674 humans 247 dogs 61 cats	Cohort	M-FECT	Humans = 14.3% Dogs = 15.3% Cats = 11.4%	Risk factors	High
**Prakobwong et al (2017) [58]**	Thailand	5347 humans 468 dogs 262 cats	Cross-sectional	M-FECT, pepsin digestion	Humans = 31.5% Dogs = 17.3% Cats = 24.4%	Risk factors	Moderate
**Pungpak et al (1997) [59]**	Thailand	1492 humans	Case-control	Microscopy	N/A	Control strategies	Moderate
**Pungpak et al (1998) [60]**	Thailand	95 humans	RCT	FECT, sedimentation	N/A	Control strategies	Some concerns
**Rangsin et al (2009) [61]**	Thailand	668 humans	Cohort	Kato thick smear, FECT	21.3%	Risk factors	Moderate
**Rattanapitoon et al (2020) [62]**	Thailand	691 humans	Cross-sectional	Mini-Parasep solvent free concentration technique	2.03%	Risk factors	Moderate
**Saengsawang et al (2013) [63]**	Thailand	684 humans	Cross-sectional	Kato-Katz	37.2%	Risk factors	Moderate
**Saengsawang et al (2024) [64]**	Thailand	457 humans	Cohort	FECT	N/A	Risk factors	Moderate
**Saiyachak et al (2016) [9]**	Laos	237 humans	Cross-sectional	Kato-Katz	54.8%	Risk factors	Moderate
**Sato et al (2015) [65]**	Laos	207 humans 970 cyprinid fish	Cross-sectional	Kato-Katz, PCR	Humans = 31.4% Fish = 1.9%	Risk factors	Moderate
**Sayasone et al (2017) [66]**	Laos	217 humans	RCT	FECT, Kato-Katz	N/A	Control strategies	Some concerns
**Sayasone et al (2007) [67]**	Laos	814 humans	Cross-sectional	Kato-Katz	58.5%	Risk factors	Moderate
**Sayasone et al (2011) [68]**	Laos	669 humans	Cross-sectional	Kato-Katz	64.3%	Risk factors	Moderate
**Sayasone et al (2015) [69]**	Laos	1313 humans	Cross-sectional	Kato-Katz	50.6%	Risk factors	Moderate
**Sayasone et al (2018) [70]**	Laos	596 humans	RCT	Kato-Katz	43.3%	Control strategies	Some concerns
**Sriamporn et al (2004) [71]**	Thailand	18393 humans	Cohort	FECT	24.5%	Risk factors	High
**Srithai et al (2021) [72]**	Thailand	250 humans	Cross-sectional	Modified Kato-Katz	24.0%	Risk factors	Moderate
**Suwannahitatorn et al (2013) [73]**	Thailand	793 humans	Cohort	Wet preparation and Kato thick smear	24.2%	Risk factors	Moderate
**Thaewnongiew et al (2014) [74]**	Thailand	3916 humans	Cross-sectional	Modified Kato-Katz	22.7%	Risk factors	Moderate
**Wattanawong et al (2021) [75]**	Thailand	126 humans	Cluster-RCT	N/A	N/A	Control strategies	Some concerns
**Wattanawong et al (2021) [7]**	Thailand	16187 humans	Cross-sectional	Kato-Katz	2.2%	Risk factors	Moderate
**Wichaiyo et al (2019) [8]**	Thailand	400 humans	Cross-sectional	Kato-Katz	N/A	Risk factors	Moderate
**Yeoh et al (2015) [76]**	Thailand	469 humans	Cross-sectional^+^	M-FECT	9.3%	Risk factors	Moderate

FECT: formalin-ethyl acetate concentration technique; N/A: not applicable; M-FECT: modified formalin-ethyl acetate concentration technique; PCR: polymerase chain reaction.

*Refers to humans unless otherwise specified.

**The Newcastle-Ottawa Scale was used for case-control, cohort, and cross-sectional studies. High refers to high quality, moderate refers to low quality. The Cochrane risk of bias tool was used for experimental studies.

#Overall prevalence not reported.

+Included a subset of the Khon Kaen Cohort.

### Risk and protective factors

Risk and protective factors associated with OV infection were examined in 51 studies. Of these studies, 40 were conducted in Thailand, eight in Laos, two in Vietnam and one in Cambodia. Identified factors included sociodemographic, environmental, and economic factors.

Diagnostic studies took place in Laos [9,10,33,55,65,68,69,77], Thailand [7,8,16,27,30,35–37,39,41–43,46,51,52,57,58,61–64,71–74,76], Vietnam [11,32] and Cambodia [48], and were conducted between 2006 and 2018. Infection identification methods varied between studies; Kato-Katz was the most frequently used diagnostic technique [7–11,29,30,33,35,38,43,47,48,55,63,65–70] followed by formalin-ethyl acetate concentration technique (FECT) [16,24,34,41,46,50–52,60,61,64,66,71]. Other methods included modified FECT [28,57,58,76], Kato thick smear [61,73], modified Kato-Katz [27,39,72,74], Mini-Parasep solvent free concentration technique [40,42,62] Polymerase Chain Reaction [65], microscopy [25,36,59], cercarial shedding [16,32,44], modified Kato thick smear [36,37], wet preparation [73], sedimentation [60] and pepsin digestion [58].

#### Sociodemographic factors.

OV age and sex distribution were reported in 34 studies. The majority of studies that examined OV infection by sex reported higher prevalence in males than females. Of the four studies that reported sex odds ratios, all found that males had significantly greater odds of OV infection than females, ranging from OR 1.78 to 9.1 (*p* < .05 in all studies) [11,27,46,52]. Five studies reported higher infection in females [27,39,58,62,68], with one study, conducted in Udon Thani Province, Thailand, reporting significantly higher OV prevalence in females than males (34.0%, 28.9% respectively, *p* < .05) [58]. There was a broad positive association between age and OV infection, although six studies reported a decline from 50 years onwards [33,36,39,42,43,51]. The highest prevalence was reported in a 2010 study in Saravane Province, Laos, where 100% of participants over 60 were OV positive [55]. Findings were consistent across regions.

One study found that body size had a positive linear relationship with OV infection [38]. The strongest relationship was reported for class II obesity (Spearman’s correlation coefficient = 0.639, *p* = .01). The relationship between being underweight and OV infection was non-significant (Spearman’s correlation coefficient = 0.301, *p* > .05) [38].

One study reported that higher income earners (>30,000 baht/month) had double the risk of reinfection (AOR 2.14, 95% CI 1.11, 4.12, *p* = .022), compared to those who earned less than 30,000 baht/month [64].

Eight studies examined the relationship between occupation and OV infection. OV prevalence in farmers or agricultural workers was reported in three studies, ranging from 9.62% to 15.46% [35,37,46]. Three studies found significantly greater odds of OV infection for agricultural workers, including labourers, fishermen, animal rearers and farmers, compared to other occupations, ranging from OR 2.21 to OR 5.16 (*p* < .05 in all studies) [29,33,57], although two studies found this relationship to be non-significant (*p* > .05) [52,64].

#### Knowledge.

Knowledge of OV was examined in eight studies between 2012 and 2018. Studies were conducted in Thailand [26,35,40,50,53,54,63] and Laos [55]. Knowledge was assessed using standardised questionnaires [26,35,40,50,53,54,63] or structured focus-group discussions [55]. Two studies examined children’s knowledge only [26,53], one study examined knowledge across all ages [50] and five studies examined adults’ knowledge only [35,41,54,55,63]. OV knowledge varied between studies. Four adult-focused studies found that the majority of participants had moderate or high OV knowledge, ranging from 60.02% to 100% [35,40,50,55]. One adult-focused study and one child-focused reported that all participants had low knowledge [26,54]. One study found that participants who did not know if PZQ prevented disease had greater odds of OV infection (AOR 2.31, 95% CI 1.40, 3.79, *p* = .001), compared to those who did believe PZQ could prevent disease [63]. One child-focused study [53] found that health literacy knowledge significantly increased the odds of good practice on liver fluke prevention; excellent access skills and absolutely corrective cognitive skills were most associated with good practice (OR 2.94, 95% CI 1.78, 4.84, *p* = .02 and OR 2.23, 95% CI 1.24, 4.01, *p* = .016 respectively) [53]. There did not appear to be any trends in age or time.

Five studies examined the relationship between education and OV [27,28,35,46,64] Two studies found that OV prevalence was higher in participants who received no education or only primary school education (8.43% to 17.6%), compared to participants who completed high school or higher education (0.5% to 3.4%) [35,46]. Similarly, one study found that participants who had primary school education only had almost twice the odds of OV infection (OR 1.8, 95% CI 1.3, 2.6) [28]. Education was found to be a protective factor for OV infection, with participants who completed secondary education having less than half the odds to OV infection compared to those who completed primary school or less (AOR 0.30, 95% CI 0.12, 0.74, *p* < .01) [27]. Another study found that secondary education or higher was a protective factor for OV infection (AOR 1.65, 95% CI 0.80, 3.42), although this result was non-significant (*p* = .172) [64]. Higher education was significantly associated with higher OV knowledge in one study (mean score 13.32 vs 12.05, *p* < .001) [40].

#### Practices.

Consumption of raw, fermented or undercooked (henceforth unsafely prepared) cyprinid fish was examined in 23 studies [8–11,16,24,26,31,33,41,48,50,52,54,56–58,61,67,73,74,78,79]. Across seven studies, prevalence of regular unsafely prepared fish consumption ranged from 36.3% to 100% [24,26,48,54,58,73,79]. Unsafely prepared fish consumption was found to be a risk factor for OV infection in 17 studies [8–11,16,25,28,31,33,41,50,52,57,61,67,73,74], although one study found this relationship was non-significant (AOR 1.05, [CI not reported] *p* = .911) [64]. The highest risk was reported by Nakbun and colleagues (2018) [50], who found that the adjusted odds of OV infection were 28.74 times greater for participants who consumed chopped raw fish salad (95% CI 3.59, 230.24, *p* < .001), compared to those who did not. One study found that family members’ consumption of raw fish increased the risk of OV infection (OR 2.47, 95% CI 1.07, 5.69, *p* = .033) [10]. Tradition was reported as a reason for consuming unsafely prepared fish dishes in three studies [58,78,79]; one study reported that to stop eating traditional raw fish dishes would contravene cultural norms and practices [78].

One study investigated predictors of unsafely prepared fish consumption. Prachaiboon and colleagues [56] found that alcohol use, limited OV knowledge, lower income, agricultural work, feeding raw fish to cats and dogs, and previous PZQ use all significantly increased the risk of unsafely prepared fish consumption, ranging from AOR 1.11 to 4.94 (*p* < .05 for all factors). Access to and understanding of health information were positively associated with reduced rates of unsafely prepared fish consumption (AOR 0.77, 95% CI 0.49, 1.19, *p* < .001 and AOR 0.99, 95% CI 0.61, 1.62, *p* < .001, respectively) [56].

One study examined the relationship between alcohol and cigarette smoking, and OV infection. Odds of OV infection were significantly greater for drinkers compared to non-drinkers (AOR 5.3, 95% CI 1.2, 23.0, *p* = .026) [76]. Smokers’ risk of OV infection was tenfold that of non-smokers (AOR 10.1, 95% CI 2.4, 41.6, *p* = .001) [76].

Two studies reported that close proximity to others with OV infection increased the risk of future infection. One study reported that close proximity to others with OV infection doubled the odds of infection (OR 2.1, 95% CI 1.5, 3.1, *p* < .05) [28]. A separate study that examined the risk for children specifically, found that having an OV-positive mother resulted in a ten-fold risk of OV infection for the child, compared to those whose mothers were OV-negative (AOR 10.45, 95%CI 3.13, 34.86, *p* < .001) [10].

#### Sanitation.

The relationship between OV infection and sanitation was examined in three studies. One study reported that the absence of a safe place to dispose waste food at home doubled the risk of OV infection (AOR 2.04, 1.10, 3.80, p = .02) [31]. Another study found that a household with ‘poor sanitation’ had almost triple the risk of OV infection, compared to those that did not (OR 2.8, 95% CI 1.9, 4.2, *p* < .001) [28]. Conversely, Sayasone and colleagues (2007) [67] reported that participants with a sanitation facility at home had one-quarter the risk of OV infection, compared to those who did not (OR = 0.26, 95% CI 0.11, 0.57, *p* < 0.001). Two studies reported that living in close proximity to a water source increased odds of OV infection, ranging from OR 2.2 [58] to OR 9.6 [28] (*p* < .001 in both studies).

#### Intermediate and reservoir hosts.

Four studies reported on risk factors associated with intermediate and reservoir hosts. Aunpromma and colleagues (2016) [25] found that cats that reportedly consumed raw fish had almost twice the risk of OV infection, compared to cats that did not (OR 1.82 95% CI 1.05, 3.16, *p* < 0.05). Having a cat at home was identified as a major risk factor for OV infection in humans, even after adjusting for fish consumption (AOR 7.0, 95% CI 1.36, 36.09, *p* < .05) [8]. Two studies examined factors that contributed to infection in aquatic snails. Studies were conducted in Thailand [44] and Vietnam [32] between 2010 and 2015. The Thailand study found that season was significantly associated with overall OV prevalence with prevalence higher in the cool-dry season (following the rainy season), compared to the hot-dry and rainy season (OR 2.07, 95% CI 1.80, 2.37, *p* < .001), although seasonal prevalence varied by year [44]. Infection increased over time, reported as 0.34% in 2010–2011, and 4.10% in 2013. These changes were positively correlated with the amount of irrigated water released [44]. Similarly, the Vietnam study found that snail habitat was significantly associated with prevalence and intensity of OV, with prevalence 13.60 times greater among snails located in irrigation canals compared to rice fields (OR 13.60, 95% CI 1.31, 141.25, *p* = .029) [32], although authors did note that this estimate was imprecise due to the very low observed prevalence among snails in rice fields (0.11%) compared to canals (1.17%) [32].

#### Medical examination.

Two studies reported on the relationship between previous helminth infection and OV infection. Both studies found that previous helminth infection significantly increased the odds of OV infection, ranging from AOR 5.64 to 8.69 (*p* < .01 in both studies) [30,31].

Six studies examined the relationship between anthelmintic use and OV infection. One study found that previous use of anthelmintic drugs halved the odds of OV infection (AOR 0.43, 95% CI 0.22-0.88, *p* < 0.05) [8]. Similarly, another study found that those with no history of parasitic treatment had more than triple the risk of OV infection compared to those who did (OR 3.3, 95% CI 0.6, 13.6, *p* = .037) [58]. However, four studies found that anthelmintic drug was associated with a significantly higher odds of having OV infection, ranging from AOR 1.4 to AOR 5.66 (*p* < .05 in all studies) [28,31,63,74].

Two studies examined the relationship between previous stool examination and OV infection. One study reported that previous stool examination was a protective factor for OV infection (AOR 0.22, 95% CI 0.10, 0.51, *p* = .001) [30], however a separate study found that previous stool examination was associated with increased odds of OV reinfection (AOR 2.47, 95% CI 1.13, 5.43, *p* = .023) [49].

### Control strategies

OV control strategies were examined in 11 studies. Studies were conducted in Laos [47,66,70] and Thailand [28,34,45,49,54,59,60,75]. All were conducted between 1989 and 2023.

The efficacy of PZQ was investigated in six human treatment studies [34,47,59,60,66,70]. Studies were conducted in regions in Laos [47,66,70] and Thailand [34,59,60] between 1989 and 2014. Five studies compared efficacy of different PZQ dosages, including single dosages, split doses, and triple doses [34,47,59,60,66]. One study examined the efficacy of PZQ compared to tribendimidine (TBD) on OV infection [70]. Follow-up periods varied between 14 and 60 days after treatment. The majority of studies reported PZQ cure rates above 90.0%, ranging from 92.0% to 99.7% [34,59,60,66,70], however one study reported a cure rate of 50% for high intensity cases that received PZQ single dose 40mg/kg [47]. TBD single dose (200mg in children aged 8–14 years, 400mg in persons aged ≥15 years) was found to have an efficacy of 93.6% [70].

Health education as a control strategy was examined in four studies. All studies were conducted in Thailand between 2017 and 2023 [45,49,54,75]. Two studies examined the impact of OV education interventions on associated knowledge, attitudes and practices (KAP) among adults [54,75]. One intervention was conducted in-person [54], and the other was web-based [75]; both targeted OV education and behavioural change in their interventions. One study assessed a school-based health intervention that focused on OV and CCA education through teacher-led individual- and group-based counselling, student, and public interest activities. Students were also encouraged to engage with their families at home [45]. All three studies achieved significant KAP improvements among the intervention group (*p* < .05), and significant differences in mean KAP scores compared to the control group (*p* < .05). The greatest difference in intervention and control knowledge was reported in the school-based study, in which intervention schools had a mean knowledge score of 66.9%, compared to control schools’ mean knowledge score of 1.8% (*p* < .001) [45]. The fourth study examined perceived need for OV and CCA education including knowledge, prevention, risk factors and risk behaviours, among schoolchildren [49]. The study found that children believed there was a “high level of need” for more education on all topics, with 58.33% of children reporting ‘highest’ need for education on prevention of CCA, OV and CCA risk factors, and family roles in prevention [49].

One study examined the effectiveness of a ‘One Health’ intervention on OV control. The study was conducted in Thailand between 2020 and 2022 [28]. The intervention included anthelmintic treatment for humans, reservoir hosts (cats and dogs), and intermediate hosts (cyprinid fish), community engagement, and health education methods. At 12-month follow-up there was a significant decline in human prevalence (from 6.0% to 0.3%, *p* < .05) and reductions in prevalence in in dogs (5.4% to 4.3%) and cyprinid fish (11.5% to 7.2%), however cat prevalence increased by 2.0% (from 6.3% to 8.3%) [28]. At 24-month follow-up, reductions were reported for all animals included in the study; differences between baseline prevalence and 24-month follow-up in humans (6.0% to 0.3%) and cyprinid fish (11.5% to 4.1%) were significant (*p* < .05) [28].

## Discussion

This systematic review summarises evidence from 59 studies on OV risk factors and control strategies in LMB. The majority of studies were conducted in Thailand (32 cross-sectional, six cohort, three cluster-RCTs, two case-control, one quasi-experimental, and one RCT), followed by Laos (eight cross-sectional, three RCT), Vietnam (two cross-sectional), and Cambodia (one cross-sectional). To the best of our knowledge, this is the first systematic review to synthesize OV risk factors and control strategies using a One Health lens. Through this synthesis we identified factors implicated in OV burden and areas of opportunity for prevention and control.

Age and male sex were found to be positively associated with OV infection, although there was some variability between studies. While the majority of studies reported higher OV prevalence in males, five reported greater prevalence among females, although this was only significant in one study [58]. It is possible that the sex distribution differences may be due to differing regional habits; as most studies were conducted in one provincial site, the generalisability of prevalence estimates may be limited. Similarly, it is possible that infection prevalence varied over time, as included studies took place between 1989 and 2021, although prevalence rates did appear to be broadly consistent.

The most apparent difference between studies was the diagnostic method, which varied considerably between studies, although not over time. The variability in diagnostic tools may be due to test availability or accessibility in different environments. Several studies reported on the sensitivity and specificity of different diagnostic tools, particularly in cases of low infection intensity [65,80,81]. Other studies have also noted that tests such as Kato-Katz may be inaccurate due to the morphological similarity between OV and minute intestinal flukes, both found in freshwater fish in the LMB [82,83]. It may be that prevalence estimates were influenced by use of different diagnostic approaches.

The most reported risk factor was consumption of unsafely prepared fish. All human studies and one animal study [25] found that unsafely prepared fish consumption significantly increased the odds of OV infection. Interestingly, one study found that lower income was associated with unsafely prepared fish consumption [56], while another study reported that lower income was associated with reduced OV risk [64]. The conflicting findings may be due to lifestyle changes among lower income workers [64]. Lower income earners often work in agricultural fields, however, as agricultural yields decline due to factors such as overfishing and damming in other areas of the Mekong River [84,85] workers may look elsewhere.

Knowledge and education –both general schooling and health-specific education– were inversely correlated with OV infection, indicating that expansion of control strategies targeting health education, particularly safe fish preparation for humans and animals, may reduce OV burden. It is worth noting that Suwannahitatorn and colleagues (2019) [78] reported that avoidance of unsafely prepared fish dishes, such as koi pla, was perceived as contravening cultural norms, which may be a barrier to behavioural change. However, other research [86] has shown it is possible for cultural or traditional practices to be modified for health purposes in an acceptable way.

Environmental factors were found to significantly impact OV burden in aquatic snails, the first intermediate host in the OV lifecycle. One study found that snail OV prevalence was highest in the cool-dry season [44]. This is particularly concerning as droughts in the LMB resulting from climate change [85] will likely prolong the season. Another factor is increased uptake of irrigation to maximise agricultural production in the LMB. [84,85], a method that was linked to increased snail OV prevalence [32]. These findings suggest that snail prevalence will increase dramatically in the coming years, which will likely increase OV infection in subsequent hosts. Techniques to reduce snail populations or minimise contact with possible OV transmission sites, such as constructing roads alongside waterways [87] are recommended. Several studies demonstrated the high efficacy of anthelmintic treatment in reducing OV infection. The majority of studies supported PZQ as an efficacious treatment for OV infection, although it is worth noting that one study reported only 50% efficacy for high intensity cases who received single dose treatment [47]. This suggests that multi-dose treatment is required for severe cases, although it is worth noting the small sample size of the study. Alternatively, the high efficacy of single dose TBD [70] suggests that it may be a suitable alternative in the future. It is worth noting that sole reliance on MDA does not prevent re-infection, or reverse biliary abnormalities that may occur in severe cases [13].

Health education interventions were shown to be effective in improving OV KAP in adults and children [7,45,54]. The success of web-based as well as in-person interventions suggests that delivery may be adapted to be suitable for different audiences to expand access to health education tools. The school-based intervention was the most successful. This may be due to the variety of activities used in the intervention, or the engaging delivery environment. Schools have previously recognised as an important vehicle for health promotion [88], and this is supported by other studies that have demonstrated the success of school-based interventions for sustained improvement in helminth KAP [89,90].

The ‘One Health’ study achieved marked reductions across definitive, reservoir, and intermediate hosts [28]. ‘One Health’ approaches have been endorsed in health literature for their multifaceted approach [91,92], which can also lead to long-term social and economic improvements [93]. The success of the ‘One Health’ approach has been demonstrated in previous OV research, such as the ‘Lawa Model’, a multicomponent intervention including anthelmintic treatment, and school- and community-based OV education, that achieved marked reductions in human and animal OV burden [84]. Given the complexity of the OV lifecycle, combined methods that utilise anthelmintic treatment for humans and animals, and education are recommended.

Literature on risk factors and control strategies in Cambodia and Vietnam were limited, with one study from Cambodia identified, and two studies from Vietnam. As a result, it is possible that our findings may not fully represent OV risk factors and existing control strategies in the LMB. The lack of studies in Cambodia and Vietnam underscores a need for greater field-based research focusing on OV risk factors and control strategies in these countries. While we included all primary research study types that met the inclusion criteria, a limitation of this review is that we only included studies published in English. As such, we may have missed relevant articles.

## Conclusion

*Opisthorchis viverrini* remains a major health issue in the Lower Mekong Basin. To address this health concern, it is important to understand the roles that intermediate hosts and reservoir hosts, environment and human behaviour play in disease transmission. It is recommended that multiple diagnostic techniques be used to provide a more accurate depiction of disease prevalence and control strategy effectiveness. In addition, anthelmintic treatment successfully treats disease, but it does not prevent reinfection. Therefore, a multi-faceted approach is needed. Health education interventions outlining safe fish preparation practices and environmental management should be utilised in partnership with anthelmintic treatment to improve immediate and long-term health outcomes. As such, it is recommended that future research on *O. viverrini* control utilises integrated approaches, such as ‘One Health’, that address multiple factors to improve health outcomes, rather than one area alone. Such research should be conducted in all Lower Mekong countries to improve health outcomes across the region.

## Supporting information

S1 Table PRISMA preferred reporting items for systematic reviews and meta-analyses checklist [18]. (PDF)

S2 Table Quality assessment results of included cross-sectional studies. (PDF)

S3 Table Quality assessment results of included case-control studies. (PDF)

S4 Table Quality assessment results of included cohort studies. (PDF)

S5 Table Risk of bias assessment for cluster-RCTs. (PDF)

S6 Table Risk of bias assessment for RCTs. (PDF)

S7 Table Studies excluded from the review. (XLSX)

S1 Text Search terms for systematic literature review. (PDF)

S2 Text Adapted Newcastle-Ottawa Scale for epidemiological studies. (PDF)

S3 Text Adapted Newcastle-Ottawa Scale for animal studies. (PDF)
