# Supplementary material for: Risk factors and control of Opisthorchis viverrini in the Lower Mekong Basin: A systematic review
Source: PLoS Negl Trop Dis. 2025 Dec 11;19(12):e0013790. doi: 10.1371/journal.pntd.0013790 (PMC12698015; doi:10.1371/journal.pntd.0013790)
Supplement: S2 Table — (PDF) [file pntd.0013790.s002.pdf]

S2 Table. Quality assessment results of included cross-sectional studies.

	**Selection**	**Comparability**	**Outcome**	**Rating**
**Araki et al (2018)[1]**	****		**	Moderate
**Aunpromma et al (2012)[2][#]**	*	**	*	Moderate
**Aunpromma et al (2016)[3][#]**	*	**	**	Moderate
**Chaiputcha et al (2015)[4]**	**		**	Moderate
**Charoensuk et al (2022)[5]**	***		**	Moderate
**Chuangchaiya et al (2019)[6]**	**		***	Moderate
**Chuangchaiya et al (2020)[7]**	**		***	Moderate
**Dao et al (2016)[8]**	**		**	Moderate
**Dao et al (2017)[9][#]**		**	**	Moderate
**Forrer et al (2012)[10]**	*	*	**	Moderate
**Haswell-Elkins et al (1991)[11][+]**	***		**	Moderate
**Kaewpitoon et al (2016)[12]**	***		**	Moderate
**Kaewpitoon et al (2012)[13]**	***		*	Moderate
**Kaewpitoon et al (2012)[14]**	***		*	Moderate

	Selection	Comparability	Outcome	Rating
**Kaewpitoon et al (2016)[15]**	***		**	Moderate
**Kaewpitoon et al (2016)[16]**	***		**	Moderate
**Kaewpitoon et al (2016)[17]**	**		**	Moderate
**Kaewpitoon et al (2018)[18]**	***		**	Moderate
**Kaewpitoon et al (2019)[19]**	***		**	Moderate
**Kitphati et al (2021)[20]⁺**	***		**	Moderate
**Laoraksawong et al (2018)[21]**	**	*	**	Moderate
**Miyamoto et al (2014)[22]**	***		**	Moderate
**Moonsan et al (2024)[23]**	**		**	Moderate
**Nakbun et al (2018)[24]**	**		**	Moderate
**Nithikathkul et al[25] (2009)**	***		**	Moderate
**Ong et al (2016)[26]**	**		**	Moderate
**Padchasuwan et al (2018)[27]**	***	**	**	High
**Phongluxa et al (2013)[28]**	***		**	Moderate

	Selection	Comparability	Outcome	Rating
**Prachaiboon et al (2021)[29]**	***		*	Moderate
**Prakobwong et al (2017)[30]**	**		**	Moderate
**Rattanapitoon et al (2020)[31][+]**	***		**	Moderate
**Saengsawang et al (2013)[32]**	*	**	**	Moderate
**Saiyachak et al (2016)[33]**	**	*	**	Moderate
**Sato et al (2011)[34][+]**	***		**	Moderate
**Sayasone et al (2007)[35]**	***		**	Moderate
**Sayasone et al (2011)[36]**	*	*	**	Moderate
**Sayasone et al (2015)[37]**	***	*	**	Moderate
**Srithai et al (2021)[38]**	**		***	Moderate
**Thaewnongiew et al (2014)[39]**	***		*	Moderate
**Wattanawong et al (2021)[40]**	**	**	**	Moderate
**Wichaiyo et al (2019)[41]**	*	*	**	Moderate
**Kopolrat et al (2020)[42][#+]**		**	**	Moderate

	Selection	Comparability	Outcome	Rating
**Yeoh et al (2015)[43]**	**		**	Moderate

*Score adjusted for non-applicable sections
#Animal study
+Epidemiological study
Quality assessment was performed using adapted versions of the Newcastle-Ottawa Scale [44, 45].

References

1. Araki H, Ong KIC, Lorphachan L, Soundala P, Iwagami M, Shibanuma A, et al. Mothers’ Opisthorchis viverrini infection status and raw fish dish consumption in Lao People’s Democratic Republic: determinants of child infection status. Trop Med Health. 2018;46:29.

2. Aunpromma S, Tangkawattana P, Papirom P, Kanjampa P, Tesana S, Sripa B, et al. High prevalence of Opisthorchis viverrini infection in reservoir hosts in four districts of Khon Kaen Province, an opisthorchiasis endemic area of Thailand. Parasitol Int. 2012 Mar;61(1):60–4.

3. Aunpromma S, Kanjampa P, Papirom P, Tangkawattana S, Tangkawattana P, Tesana S, et al. PREVLENCE AND RISK FACTORS FOR OPISTHORCHIS VIVERRINI INFECTION AMONG CATS AND DOGS IN SIX DISTRICTS SURROUNDING THE UBOLRATANA DAM, AN ENDEMIC AREA FOR HUMAN OPISTHORCHIASIS IN NORTHEASTERN THAILAND. Southeast Asian J Trop Med Public Health. 2016 Nov;47(6):1153–9.

4. Chaiputcha K, Promthet S, Bradshaw P. Prevalence and Risk Factors for Infection by Opisthorchis viverrini in an Urban Area of Mahasarakham Province, Northeast Thailand. Asian Pac J Cancer Prev. 2015;16(10):4173–6.

5. Charoensuk L, Ribas A, Chedtabud K, Prakobwong S. Infection rate of Opisthorchis viverrini metacercariae in cyprinoid fish from the markets and its association to human opisthorchiasis in the local community in the Northeast Thailand. Acta Tropica. 2022 Jan 1;225:106216.

6. Chuangchaiya S, Laoprom N, Idris ZM. Prevalence and associated risk factors of Opisthorchis viverrini infections in rural communities along the Nam Kam River of Northeastern Thailand. Trop Biomed. 2019 Mar 1;36(1):81–93.

7. Chuangchaiya S, Navanesan S, Jaichuang S, Rahim M, Idris ZM. Current prevalence of Opisthorchis viverrini infection and associated risk factors in Nakhon Phanom Province, Northeastern Thailand. Trop Biomed. 2020 Dec 1;37(4):986–99.

8. Dao TTH, Bui TV, Abatih EN, Gabriël S, Nguyen TTG, Huynh QH, et al. Opisthorchis viverrini infections and associated risk factors in a lowland area of Binh Dinh Province, Central Vietnam. Acta Tropica. 2016 May 1;157:151–7.

9. Dao HTT, Dermauw V, Gabriël S, Suwannatrai A, Tesana S, Nguyen GTT, et al. Opisthorchis viverrini infection in the snail and fish intermediate hosts in Central Vietnam. Acta Tropica. 2017 Jun 1;170:120–5.

10. Forrer A, Sayasone S, Vounatsou P, Vonghachack Y, Bouakhasith D, Vogt S, et al. Spatial distribution of, and risk factors for, Opisthorchis viverrini infection in southern Lao PDR. PLoS Negl Trop Dis. 2012;6(2):e1481.

11. Haswell-Elkins MR, Elkins DB, Sithithaworn P, Treesarawat P, Kaewkes S. Distribution patterns of Opisthorchis viverrini within a human community. Parasitology. 1991 Aug;103 Pt 1:97–101.

12. Kaewpitoon SJ, Wakkuwattapong R, Rujirakul R, Wakkuwattapong P, Matrakool L, Tongtawee T, et al. Opisthorchis viverrini infection among people in the border areas of three provinces, northeast of thailand. Asian Pacific Journal of Cancer Prevention. 2016;17(6):2973–7.

13. Kaewpitoon SJ, Rujirakul R, Kaewpitoon N. Prevalence of Opisthorchis viverrini infection in Nakhon Ratchasima province, Northeast Thailand. Asian Pac J Cancer Prev. 2012;13(10):5245–9.

14. Kaewpitoon SJ, Rujirakul R, Ueng-Arporn N, Matrakool L, Namwichaisiriku N, Churproong S, et al. Community-based cross-sectional study of carcinogenic human liver fluke in elderly from Surin province, Thailand. Asian Pac J Cancer Prev. 2012;13(9):4285–8.

15. Kaewpitoon SJ, Rujirakul R, Wakkuwattapong P, Matrakool L, Tongtawee T, Panpimanmas S, et al. Overweight Relation to Liver Fluke Infection among Rural Participants from 4 Districts of Nakhon Ratchasima Province, Thailand. Asian Pacific Journal of Cancer Prevention. 2016;17(5):2565–71.

16. Kaewpitoon SJ, Rujirakul R, Loyd RA, Panpimanmas S, Matrakool L, Tongtawee T, et al. Re-Examination of Opisthorchis viverrini in Nakhon Ratchasima Province, Northeastern Thailand, Indicates Continued Needs for Health Intervention. Asian Pac J Cancer Prev. 2016;17(1):231–4.

17. Kaewpitoon SJ, Kaewpitoon N, Rujirakul R, Wakkuwattapong P, Matrakul L, Tongtawee T, et al. Nurses and Television as Sources of Information Effecting Behavioral Improvement Regarding Liver Flukes in Nakhon Ratchasima Province, Thailand. Asian Pac J Cancer Prev. 2016;17(3):1097–102.

18. Kaewpitoon SJ, Sangwalee W, Kujapun J, Norkaew J, Wakkhuwatapong P, Chuatanam J, et al. Opisthorchis viverrini infection among migrant workers in Nakhon Ratchasima province, Thailand, indicates continued need for active surveillance. Trop Biomed. 2018 Jun 1;35(2):453–63.

19. Kaewpitoon SJ, Ponphimai S, Pechdee P, Thueng-In K, Khiaowichit J, Meererksom T, et al. The prevalence of intestinal helminth infection in rural subdistricts of northeastern Thailand. Trop Biomed. 2019 Mar 1;36(1):152–64.

20. Kitphati R, Watanawong O, Wongsaroj T, Nithikathkul C. National Program of Opisthorchiasis in Thailand; Situation and Policy Strategy. International Journal of Geoinformatics. 2021 Apr 2;17(2):61–8.

21. Laoraksawong P, Sanpool O, Rodpai R, Thanchomnang T, Kanarkard W, Maleewong W, et al. Current high prevalences of Strongyloides stercoralis and Opisthorchis viverrini infections in rural communities in northeast Thailand and associated risk factors. BMC Public Health. 2018 Jul 31;18(1):940.

22. Miyamoto K, Kirinoki M, Matsuda H, Hayashi N, Chigusa Y, Sinuon M, et al. Field survey focused on Opisthorchis viverrini infection in five provinces of Cambodia. Parasitology International. 2014 Apr 1;63(2):366–73.

23. Moonsan S, Songserm N, Phitchayapirath P. Teaching Strategies to Enhance Knowledge and Understanding of Opisthorchis viverrini and Cholangiocarcinoma in Thailand: Lessons for Asian Countries. J Cancer Educ. 2024 Sep 2;

24. Nakbun S, Thongkrajai P, Nithikathkul C. Risk factors for Opisthorchis viverrini infection in Nakhon Phanom, Thailand, where the infection is highly endemic. Asian Biomedicine. 2018;12(1):45–51.

25. Nithikathkul C, Pumidonming W, Wannapinyosheep S, Tesana S, Chaiprapathong S, Wongsawad C. Opisthorchis viverrini infection in minute intestinal fluke endemic areas of Chiang Mai Province, Thailand. Asian Biomedicine. 2009;3(2):187–91.

26. Ong X, Wang YC, Sithithaworn P, Namsanor J, Taylor D, Laithavewat L. Uncovering the Pathogenic Landscape of Helminth (Opisthorchis viverrini) Infections: A Cross-Sectional Study on Contributions of Physical and Social Environment and Healthcare Interventions. PLoS Negl Trop Dis. 2016 Dec;10(12):e0005175.

27. Padchasuwan N, Banchonhattakit P, Kaewpitoon N. Health literacy associated with liver fluke prevention and control among secondary school students in Northeast Thailand. Suranaree Journal of Science & Technology. 2018;25(3).

28. Phongluxa K, Xayaseng V, Vonghachack Y, Akkhavong K, van Eeuwijk P, Odermatt P. Helminth infection in southern Laos: high prevalence and low awareness. Parasites & vectors. 2013 Nov 14;6(1):328.

29. Prachaiboon, T, Banchobhattakit, P, Rattanapitoon, NK, Phimha, S. Health literacy associated with raw cyprinoid fish consumption in Northeastern Thailand. Medico-Legal Update. 2021;21(1).

30. Prakobwong S, Gunnula W, Chaipibool S, Nimala B, Sangthopo J, Sirivetthumrong N, et al. Epidemiology of Opisthorchis viverrini in an endemic area of Thailand, an integrative approach. Helminthologia. 2017 Dec;54(4):298–306.

31. Rattanapitoon SK, Pechdee P, Boonsuya A, Meererksom T, Wakkhuwatapong P, Leng M, et al. Prevalence and intensity of helminths among inhabitants of the Chi River and Lahanna water reservoir areas of Northeastern Thailand. Trop Biomed. 2020 Sep 1;37(3):730–43.

32. Saengsawang P, Promthet S, Bradshaw P. Infection with Opisthorchis viverrini and use of praziquantel among a working-age population in northeast Thailand. Asian Pac J Cancer Prev. 2013;14(5):2963–6.

33. Saiyachak K, Tongsotsang S, Saenrueang T, Moore MA, Promthet S. Prevalence and Factors Associated with Opisthorchis viverrini Infection in Khammouane Province, Lao PDR. Asian Pac J Cancer Prev. 2016;17(3):1589–93.

34. Sato M, Pongvongsa T, Sanguankiat S, Yoonuan T, Kobayashi J, Boupha B, et al. Patterns of trematode infections of Opisthorchis viverrini (Opisthorchiidae) and Haplorchis taichui (Heterophyidae) in human populations from two villages in Savannakhet Province, Lao PDR. J Helminthol. 2015 Jul;89(4):439–45.

35. Sayasone S, Odermatt P, Phoumindr N, Vongsaravane X, Sensombath V, Phetsouvanh R, et al. Epidemiology of Opisthorchis viverrini in a rural district of southern Lao PDR. Trans R Soc Trop Med Hyg. 2007 Jan;101(1):40–7.

36. Sayasone S, Mak TK, Vanmany M, Rasphone O, Vounatsou P, Utzinger J, et al. Helminth and intestinal protozoa infections, multiparasitism and risk factors in Champasack province, Lao People's Democratic Republic. PLoS Negl Trop Dis. 2011 Apr 12;5(4):e1037.

37. Sayasone S, Utzinger J, Akkhavong K, Odermatt P. Multiparasitism and intensity of helminth infections in relation to symptoms and nutritional status among children: A cross-sectional study in southern Lao People's Democratic Republic. Acta Tropica. 2015 Jan 1;141:322–31.

38. Srithai C, Chuangchaiya S, Jaichuang S, Idris ZM. Prevalence of Opisthorchis viverrini and Its Associated Risk Factors in the Phon Sawan District of Nakhon Phanom Province, Thailand. Iran J Parasitol. 2021 Sep;16(3):474–82.

39. Thaewnongiew K, Singthong S, Kutchamart S, Tangsawad S, Promthet S, Sailugkum S, et al. Prevalence and risk factors for Opisthorchis viverrini infections in upper Northeast Thailand. Asian Pac J Cancer Prev. 2014;15(16):6609–12.

40. Wattanawong O, Iamsirithaworn S, Kophachon T, Nak-ai W, Wisetmora A, Wongsaroj T, et al. Current status of helminthiases in Thailand: A cross-sectional, nationwide survey, 2019. Acta Tropica. 2021 Nov 1;223:106082.

41. Wichaiyo W, Parnsila W, Chaveepojnkamjorn W, Sripa B. Predictive risk factors towards liver fluke infection among the people in Kamalasai District, Kalasin Province, Thailand. SAGE Open Med. 2019;7:2050312119840201.

42. Kopolrat K, Sithithaworn P, Kiatsopit N, Namsanor J, Laoprom N, Tesana S, et al. Influence of Water Irrigation Schemes and Seasonality on Transmission Dynamics of Opisthorchis viverrini in the Snail Intermediate Host, Bithynia siamensis goniomphalos in Rice Paddy Fields in Northeast Thailand. Am J Trop Med Hyg. 2020 Jul;103(1):276–86.

43. Yeoh KW, Promthet S, Sithithaworn P, Kamsa-Ard S, Parkin DM. Re-examination of Opisthorchis viverrini Infection in Northeast Thailand. Asian Pac J Cancer Prev. 2015;16(8):3413–8.

44. Wells GA, Shea B, O'Connell D, Peterson J, Welch V, Losos M, et al. The Newcastle-Ottawa Scale (NOS) for assessing the quality of nonrandomised studies in meta-analyses. 2000. https://www.ohri.ca/programs/clinical_epidemiology/oxford.asp

45. Herzog R, Álvarez-Pasquin MJ, Díaz C, Del Barrio JL, Estrada JM, Gil Á. Are healthcare workers' intentions to vaccinate related to their knowledge, beliefs and attitudes? a systematic review. BMC Public Health. 2013 Feb 19;13:154.
