# Supplementary material for: Risk factors and control of Opisthorchis viverrini in the Lower Mekong Basin: A systematic review
Source: PLoS Negl Trop Dis. 2025 Dec 11;19(12):e0013790. doi: 10.1371/journal.pntd.0013790 (PMC12698015; doi:10.1371/journal.pntd.0013790)
Supplement: S3 Table — (PDF) [file pntd.0013790.s003.pdf]

**S3 Table. Quality assessment results of included case-control studies.**

| | **Selection** | **Comparability** | **Exposure** | **Quality rating** |
|---|---|---|---|---|
| **Chudthaisong et al (2015)[1]** | **** | | ** | Moderate |
| **Pungpak et al (1997) [2]** | *** | * | ** | Moderate |

Quality assessment was performed using the Newcastle-Ottawa Scale [3].

## References

1. Chudthaisong N, Promthet S, Bradshaw P. Risk factors for Opisthorchis viverrini Infection in Nong Khai Province, Thailand. Asian Pac J Cancer Prev. 2015;16(11):4593–6.

2. Pungpak S, Viravan C, Radomyos B, Chalermrut K, Yemput C, Plooksawasdi W, et al. Opisthorchis viverrini Infection in Thailand: Studies on the Morbidity of the Infection and Resolution Following Praziquantel Treatment. The American Journal of Tropical Medicine and Hygiene. 1997 Mar 1;56(3):311–4.

3. Wells GA, Shea B, O'Connell D, Peterson J, Welch V, Losos M, et al. The Newcastle-Ottawa Scale (NOS) for assessing the quality of nonrandomised studies in meta-analyses. 2000. https://www.ohri.ca/programs/clinical_epidemiology/oxford.asp
