# Supplementary material for: Risk factors and control of Opisthorchis viverrini in the Lower Mekong Basin: A systematic review
Source: PLoS Negl Trop Dis. 2025 Dec 11;19(12):e0013790. doi: 10.1371/journal.pntd.0013790 (PMC12698015; doi:10.1371/journal.pntd.0013790)
Supplement: S4 Table — (PDF) [file pntd.0013790.s004.pdf]

**S4 Table. Quality assessment results of included cohort studies.**

| | Selection | Comparability | Outcome | Quality rating |
|---|---|---|---|---|
| **Charoensuk et al (2024)[1]** | **** | * | *** | High |
| **Prakobwong & Suwannatrai (2020)[2]** | **** | * | ** | High |
| **Rangsin et al (2009)[3]** | *** | | ** | Moderate |
| **Saensawang et al (2024)[4]** | *** | * | ** | Moderate |
| **Sriamporn et al (2004)[5]** | **** | * | ** | High |
| **Suwannahitatorn et al (2013)[6]** | **** | | ** | Moderate |

Quality assessment was performed using the Newcastle-Ottawa Scale [7].

**References**


1. Charoensuk L, Chedtabud K, Chaipibool S, Laothong U, Suwannatrai A, Pinlaor S, et al. Integrated One-Health approach for prevention and control of Opisthorchis viverrini infection in rural Thailand: a 3-year study. Parasitol Res. 2024 Jun 28;123(7):258.

2. Prakobwong S, Suwannatrai K. Reduction of Reinfection Rates with Opisthorchis viverrini through a Three-Year Management Program in Endemic Areas of Northeastern Thailand. Korean J Parasitol. 2020;58(5):527–35.

3. Rangsin R, Mungthin M, Taamasri P, Mongklon S, Aimpun P, Naaglor T, et al. Incidence and Risk Factors of Opisthorchis viverrini Infections in a Rural Community in Thailand. The American Journal of Tropical Medicine and Hygiene. 2009 Jul 1;81(1):152–5.

4. Saengsawang P, Buakate P. Re- Infection Rate of Opisthorchis Viverrini Five Years After Treatment with Praziquantel in High-Risk Area: A Community-Based Study. Asian Pac J Cancer Prev. 2024 Aug 1;25(8):2679–84.

5. Sriamporn S, Pisani P, Pipitgool V, Suwanrungruang K, Kamsa-ard S, Parkin DM. Prevalence of Opisthorchis viverrini infection and incidence of cholangiocarcinoma in Khon Kaen, Northeast Thailand. Tropical Medicine & International Health. 2004;9(5):588–94.

6. Suwannahitatorn P, Klomjit S, Naaglor T, Taamasri P, Rangsin R, Leelayoova S, et al. A follow-up study of Opisthorchis viverrini infection after the implementation of control program in a rural community, central Thailand. Parasit Vectors. 2013 Jun 20;6:188.

7. Wells GA, Shea B, O'Connell D, Peterson J, Welch V, Losos M, et al. The Newcastle-Ottawa Scale (NOS) for assessing the quality of nonrandomised studies in meta-analyses. 2000. https://www.ohri.ca/programs/clinical_epidemiology/oxford.asp
