# Supplementary material for: Risk factors and control of Opisthorchis viverrini in the Lower Mekong Basin: A systematic review
Source: PLoS Negl Trop Dis. 2025 Dec 11;19(12):e0013790. doi: 10.1371/journal.pntd.0013790 (PMC12698015; doi:10.1371/journal.pntd.0013790)
Supplement: S5 Table — (PDF) [file pntd.0013790.s005.pdf]

S5 Table. Risk of bias assessment for cluster-RCTs.

	**Domain 1a: randomisation**				**Domain 1b: recruitment**				**Domain 2: deviations from intended interventions**								**Domain 3: missing outcome data**					**Domain 4: measurement of the outcome**							**Domain 5: selection of the reported result**				**Overall bias**
**Question No.**	**1**	**2**	**3**	**Result**	**1**	**2**	**3**	**Result**	**1**	**2**	**3**	**4**	**5**	**6**	**7**	**Result**	**1**	**2**	**3**	**4**	**Result**	**1**	**2**	**3a**	**3b**	**4**	**5**	**Result**	**1**	**2**	**3**	**Result**	**Result**
**Bukkhunthod et al (2020)[1]**	Y	PY	N	Low	Y	NA	N	Low	PY	PY	NI	NA	NA	Y	NA	Some concerns	Y	NA	NA	NA	Low	N	PN	Y	Y	PN	NA	Low	NI	PN	PN	Some concerns	Some concerns
**Laithavewat et al (2020)[2]**	Y	NI	N	Some concerns	N	N	PN	Low	Y	Y	NI	NA	NA	Y	NA	Some concerns	Y	NA	NA	NA	Low	N	PN	Y	Y	N	NA	Low	NI	PN	PN	Some concerns	Some concerns
**Wattanawong et al (2021)[3]**	Y	NI	PN	Some concerns	N	N	N	Low	Y	Y	NI	NA	NA	Y	NA	Some concerns	Y	NA	NA	NA	Low	N	PN	PY	PY	PN	NA	Low	NI	PN	PN	Some concerns	Some concerns

Y, Yes; N, No; NA, Not Applicable; NI, No Information; PN, Probably No; PY, Probably Yes.
Risk of bias assessment was performed using the Cochrane Risk of Bias tool [4].

References

1. Bukkhunthod P, Meererksom T, Pechdee P, Ponphimai S, Khiaowichit J, Kaewpitoon N, et al. Animation as Supplementary Learning Material About Carcinogenic Liver Fluke in Classes for Primary Schoolchildren. J Canc Educ. 2020 Feb 1;35(1):14–21.

2. Laithavewat L, Grundy-Warr C, Khuntikeo N, Andrews RH, Petney TN, Yongvanit P, et al. Analysis of a school-based health education model to prevent opisthorchiasis and cholangiocarcinoma in primary school children in northeast Thailand. Global Health Promotion. 2020 Mar 1;27(1):15–23.

3. Wattanawong O, Prachaiboon T, Meererksom T, Rattanapitoon NK, Rattanapitoon SK, Banchonhattakit P, et al. OVCCA Web Application as Supplementary Material to Facilitate Health Literacy Regarding Carcinogenic Human Liver Fluke: A Randomized Controlled Trial in Thailand. Asian Pac J Cancer Prev. 2021 Sep 1;22(9):3045–52.

4. Higgins JPT, Thomas J, Chandler J, Cumpston M, Li T, Page MJ, et al. Cochrane Handbook for Systematic Reviews of Interventions. Vol. version 6.3 (updated February 2022). 2022.
