# Supplementary material for: Risk factors and control of Opisthorchis viverrini in the Lower Mekong Basin: A systematic review
Source: PLoS Negl Trop Dis. 2025 Dec 11;19(12):e0013790. doi: 10.1371/journal.pntd.0013790 (PMC12698015; doi:10.1371/journal.pntd.0013790)
Supplement: S6 Table — (PDF) [file pntd.0013790.s006.pdf]

S6 Table. Risk of bias assessment for RCTs.

	**Domain 1: randomisation**				**Domain 2: deviations from intended interventions**								**Domain 3: missing outcome data**					**Domain 4: measurement of the outcome**						**Domain 5: selection of the reported result**				**Overall bias**
	**1**	**2**	**3**	**Result**	**1**	**2**	**3**	**4**	**5**	**6**	**7**		**1**	**2**	**3**	**4**		**1**	**2**	**3**	**4**	**5**		**1**	**2**	**3**		
**Lovis et al (2012)[1]**	Y	Y	N	Low	PN	N	NA	NA	NA	PY	NA	Low	N	N	PN	NA	Low	N	PN	N	NA	NA	Low	NI	N	PN	Some concerns	Some concerns
**Panithanang et al (2018)[2]**	NI	NI	NI	Some concerns	Y	Y	PN	NA	NA	N	PN	Some concerns	NA	NA	NA	NA	Low	N	PN	Y	PN	NA	Low	N	N	N	Some concerns	Some concerns
**Pungpak et al (1998)[3]**	Y	NI	PN	Some concerns	NI	NI	NI	NA	NA	NI	PN	Some concerns	NA	NA	NA	NA	Low	N	PN	NI	PN	NA	Low	N	PN	PN	Some concerns	Some concerns
**Sayasone et al (2017)[4]**	Y	Y	PN	Low	N	PN	NA	NA	NA	Y	NA	Low	NA	NA	NA	NA	Low	N	PN	PN	NA	NA	Low	NI	PN	PN	Some concerns	Some concerns
**Sayasone et al (2018)[5]**	Y	PY	PN	Low	Y	Y	PN	NA	NA	Y	NA	Low	Y	Y	NA	NA	Low	N	PN	Y	PN	NA	Low	NI	N	PN	Some concerns	Some concerns

Y, Yes; N, No; NA, Not Applicable; NI, No Information; PN, Probably No; PY, Probably Yes.
Risk of bias assessment was performed using the Cochrane Risk of Bias tool [6].

References

1. Lovis L, Mak TK, Phongluxa K, Aye Soukhathammavong P, Vonghachack Y, Keiser J, et al. Efficacy of praziquantel against Schistosoma mekongi and Opisthorchis viverrini: a randomized, single-blinded dose-comparison trial. PLoS Negl Trop Dis. 2012;6(7):e1726.

2. Panithanang B, Srithongklang W, Kompor P, Pengsaa P, Kaewpitoon N, Wakkhuwattapong P, et al. The Effect of Health Behavior Modification Program for Liver Fluke Prevention among the Risk Group in Rural Communities, Thailand. Asian Pac J Cancer Prev. 2018;19(9):2673–80.

3. Pungpak S, Radomyos P, Radomyos BE, Schelp FP, Jongsuksuntigul P, Bunnag D. Treatment of Opisthorchis viverrini and intestinal fluke infections with Praziquantel. Southeast Asian J Trop Med Public Health. 1998 Jun;29(2):246–9.

4. Sayasone S, Meister I, Andrews JR, Odermatt P, Vonghachack Y, Xayavong S, et al. Efficacy and Safety of Praziquantel Against Light Infections of Opisthorchis viverrini: A Randomized Parallel Single-Blind Dose-Ranging Trial. Clin Infect Dis. 2017 Feb 15;64(4):451–8.

5. Sayasone S, Keiser J, Meister I, Vonghachack Y, Xayavong S, Senggnam K, et al. Efficacy and safety of tribendimidine versus praziquantel against Opisthorchis viverrini in Laos: an open-label, randomised, non-inferiority, phase 2 trial. Lancet Infect Dis. 2018 Feb;18(2):155–61.

6. Higgins JPT, Thomas J, Chandler J, Cumpston M, Li T, Page MJ, et al. Cochrane Handbook for Systematic Reviews of Interventions. Vol. version 6.3 (updated February 2022). 2022.
