# Supplementary material for: Risk factors and control of Opisthorchis viverrini in the Lower Mekong Basin: A systematic review
Source: PLoS Negl Trop Dis. 2025 Dec 11;19(12):e0013790. doi: 10.1371/journal.pntd.0013790 (PMC12698015; doi:10.1371/journal.pntd.0013790)
Supplement: S1 Text — (PDF) [file pntd.0013790.s008.pdf]

S1 Text. Search terms for systematic literature review.

Viverrini [Title] OR Opisthorchis [Title] OR Opisthorchiasis [Title] OR Helminth*[Title] OR liver fluke [Title] NOT Hamster[Title]

AND

Viverrini[Title/Abstract]

- AND
- Burden [Title/Abstract] OR
- Prevalence [Title/Abstract] OR
- Intensity [Title/Abstract] OR
- Distribution [Title/Abstract] OR
- Epidemiology [Title/Abstract] OR
- Infection rate [Title/Abstract] OR
- Intervention* [Title/Abstract] OR
- Risk factor* [Title/Abstract] OR
- Control [Title/Abstract] OR
- Transmission [Title/Abstract] OR
- Treatment [Title/Abstract] OR
- Drug administration [Title/Abstract] OR
- Praziquantel [Title/Abstract] OR
- Reservoir host* [Title/Abstract] OR
- Definitive host* [Title/Abstract] OR
- Cat* [Title/Abstract] OR
- Dog* [Title/Abstract] OR
- Fish [Title/Abstract] OR
- Snail [Title/Abstract] OR
- RCT [Title/Abstract] OR
- Trial [Title/Abstract]

PubMed Advanced search input:

(Viverrini[Title] OR Opisthorchis[Title] OR Opisthorchiasis[Title] OR Helminth*[Title] OR liver fluke [Title] NOT Hamster[Title]) AND Viverrini[Title/Abstract] AND (Burden[Title/Abstract] OR Prevalence[Title/Abstract] OR Intensity[Title/Abstract] OR Distribution[Title/Abstract] OR Epidemiology[Title/Abstract] OR Infection rate[Title/Abstract] OR Intervention*[Title/Abstract] OR Risk factor*[Title/Abstract] OR Control[Title/Abstract] OR Transmission[Title/Abstract] OR Treatment[Title/Abstract] OR Drug[Title/Abstract] OR Praziquantel[Title/Abstract] OR Reservoir[Title/Abstract] OR Definitive[Title/Abstract] OR Cat[Title/Abstract] OR Cats[Title/Abstract] OR Dog[Title/Abstract] OR Dogs[Title/Abstract] OR RCT[Title/Abstract] OR Trial[Title/Abstract] OR WASH[Title/Abstract] OR Fish[Title/Abstract] OR Snail*[Title/Abstract] OR RCT[Title/Abstract] OR Trial[Title/Abstract])

SCOPUS Advanced search input:

(TITLE ("Viverrini") OR TITLE ("Opisthorchis") OR TITLE ("Opisthorchiasis") OR TITLE ("Helminth") OR TITLE("liver fluke") AND NOT TITLE("HAMSTER")) AND TITLE-ABS-KEY-AUTH ("Viverrini") AND (TITLE-ABS-KEY-AUTH ("Prevalence") OR TITLE-ABS-KEY-AUTH ("Intensity") OR TITLE-ABS-KEY-AUTH ("Distribution") OR TITLE-ABS-KEY-AUTH ("Epidemiology") OR TITLE-ABS-KEY-AUTH ("Infection rate") OR TITLE-ABS-KEY-AUTH ("Intervention") OR TITLE-ABS-KEY-AUTH ("Interventions") OR TITLE-ABS-KEY-AUTH ("Risk factor") OR TITLE-ABS-KEY-AUTH ("Risk factors") OR TITLE-ABS-KEY-AUTH ("Control") OR TITLE-ABS-KEY-AUTH ("Transmission") OR TITLE-ABS-KEY-AUTH ("Treatment") OR TITLE-ABS-KEY-AUTH ("Drug administration") OR TITLE-ABS-KEY-AUTH ("Praziquantel") OR TITLE-ABS-KEY-AUTH ("Reservoir") OR TITLE-ABS-KEY-AUTH ("Definitive") OR TITLE-ABS-KEY-AUTH ("Cat") OR TITLE-ABS-KEY-AUTH ("Cats") OR TITLE-ABS-KEY-AUTH ("Dog") OR TITLE-ABS-KEY-AUTH ("Dogs") OR TITLE-ABS-KEY-AUTH ("Fish") OR TITLE-ABS-KEY-AUTH ("Snail") OR TITLE-ABS-KEY-AUTH ("Snails") OR TITLE-ABS-KEY-AUTH ("RCT") OR TITLE-ABS-KEY-AUTH ("trial"))
