# Supplementary material for: Risk factors and control of Opisthorchis viverrini in the Lower Mekong Basin: A systematic review
Source: PLoS Negl Trop Dis. 2025 Dec 11;19(12):e0013790. doi: 10.1371/journal.pntd.0013790 (PMC12698015; doi:10.1371/journal.pntd.0013790)
Supplement: S3 Text — (PDF) [file pntd.0013790.s010.pdf]

S3 Text. Adapted Newcastle-Ottawa Scale for animal studies.

This quality assessment scale for animal studies has been adapted from the original Newcastle-Ottawa Scale developed for non-randomised studies [1] and the adapted cross-sectional study version by Herzog et al. [2].

Selection: (Maximum 3 stars)

1) Sample size:
    a) Justified and satisfactory. *
    b) Not justified.
2) Ascertainment of the exposure (risk factor):
    a) Validated measurement tool. **
    b) Non-validated measurement tool, but the tool is available or described.*
    c) No description of the measurement tool.
    d) N/A

Comparability: (Maximum 2 stars)

1) The subjects in different outcome groups are comparable, based on the study design or analysis. Confounding factors are controlled.
    a) The study controls for the most important factor (select one). *
    b) The study control for any additional factor. *
    c) The study only provides information for one outcome group (e.g. only presents data on positive cases) or does not provide analyses for possible confounding factors
    d) N/A

Outcome: (Maximum 3 stars)

1) Assessment of the outcome:
    a) Independent blind assessment. **
    b) Independent assessment. *
    c) Record linkage. **
    d) Self report. *
    e) No description.
    f) N/A – study does not measure a specific outcome
2) Statistical test:
    a) The statistical test used to analyse the data is clearly described and appropriate, and the measurement of the association is presented, including confidence intervals and the probability level (p value) where relevant. *
    b) The statistical test is not appropriate, not described or incomplete.
    c) N/A

References

1. Wells GA, Shea B, O'Connell D, Peterson J, Welch V, Losos M, et al. The Newcastle-Ottawa Scale (NOS) for assessing the quality of nonrandomised studies in meta-analyses. 2000. https://www.ohri.ca/programs/clinical_epidemiology/oxford.asp

2. Herzog R, Álvarez-Pasquin MJ, Díaz C, Del Barrio JL, Estrada JM, Gil Á. Are healthcare workers' intentions to vaccinate related to their knowledge, beliefs and attitudes? a systematic review. BMC Public Health. 2013 Feb 19;13:154.
